# ENGAGING COLLEGE STUDENTS AND LIFE-LONG LEARNERS WITH INTERGENERATIONAL JUSTICE ISSUES

**DOI:** 10.1093/geroni/igad104.2076

**Published:** 2023-12-21

**Authors:** Leslie Hasche, Carson De Fries, Ashley Taeckens-Seabaugh, Briana Kohlbrenner, Hannah Decker, Asia Cutforth

**Affiliations:** University of Denver, Denver, Colorado, United States; University of Denver, Denver, Colorado, United States; University of Denver, Denver, Colorado, United States; University of Denver, Denver, Colorado, United States; University of Denver, Denver, Colorado, United States; University of Denver, Denver, Colorado, United States

## Abstract

Generational conflict and equity issues are often hot topics in popular media that expand across issues of politics, government budgets, environment, community-planning, health care, and employment. While increasing media attention highlights the need to reframe the way we talk about these topics to combat ageism, explicit academic exploration of the ways theories of social justice that consider impacts across generations and time are also needed. With the aim to provide an overview of an innovative course on intergenerational justice, we will showcase how different theories and educational activities are used to engage both graduate students and life-long learners. For over nine years, versions of this course have been offered in graduate programs, in intergenerational learning courses, and in life-long learning trainings and public events for older adults. The course frames these contemporary issues (i.e., politics, ecological justice, health care) by exploring the issue of justice between age groups, which is called intergenerational justice. Additionally, the contemporary issues will also show how considerations for intragenerational justice (i.e., injustices experienced within age groups), need to be considered within the debates of generational equity and conflict. Examples of learning activities and strategies to integrate intergenerational learning opportunities to combat attitudes about aging will be shared from both instructor and former student perspectives. Thus, we hope to demonstrate how intergenerational course topics may be a fruitful focus of gerontological education efforts to engage students, to disrupt generational conflict and ageist narratives, and to offer opportunities for intergenerational connections for problem-solving on contemporary issues.

